# Phenotypic and genotypic characterization of HMB-3, a metallo-beta-lactamase from *Pseudomonas asiatica*

**DOI:** 10.1093/jac/dkag148

**Published:** 2026-05-06

**Authors:** Jacqueline Findlay, John Michael Shaw, Patrice Nordmann, Philip Hinchliffe, Evelin Bucheli Laffer, Yannick Born, Helena M B Seth-Smith, Adrian Egli, Niels Pfennigwerth, James Spencer, Laurent Poirel

**Affiliations:** Medical and Molecular Microbiology, Faculty of Science and Medicine, University of Fribourg, Fribourg, Switzerland; School of Cellular and Molecular Medicine, University of Bristol, Bristol, UK; Medical and Molecular Microbiology, Faculty of Science and Medicine, University of Fribourg, Fribourg, Switzerland; Swiss National Reference Center for Emerging Antibiotic Resistance (NARA), University of Fribourg, Fribourg, Switzerland; School of Cellular and Molecular Medicine, University of Bristol, Bristol, UK; Department of Infectious Diseases and Hospital Hygiene, Kantonsspital Aarau AG, Aarau, Switzerland; Institute for Laboratory Medicine, Kantonsspital Aarau, Aarau, Switzerland; Institute of Medical Microbiology, University of Zurich, Zurich, Switzerland; Institute of Medical Microbiology, University of Zurich, Zurich, Switzerland; German National Reference Centre for Multidrug-Resistant Gram-Negative Bacteria, Department of Medical Microbiology, Ruhr-University Bochum, Bochum, Germany; School of Cellular and Molecular Medicine, University of Bristol, Bristol, UK; Medical and Molecular Microbiology, Faculty of Science and Medicine, University of Fribourg, Fribourg, Switzerland; Swiss National Reference Center for Emerging Antibiotic Resistance (NARA), University of Fribourg, Fribourg, Switzerland

## Abstract

**Objectives:**

We describe the identification and characterization of the HMB-3 variant, produced by a *P. asiatica* isolate from a patient in Switzerland.

**Materials and methods:**

A carbapenem-resistant *P. asiatica* isolate was sent to the Swiss National Reference Center for Emerging Antibiotic Resistance for investigation. Antibiotic susceptibility testing was performed according to CLSI guidelines. WGS was performed on Illumina and Oxford Nanopore platforms. The *bla*_HMB_ alleles were cloned into the pCR-Blunt II-TOPO plasmid. Site-directed mutagenesis was performed on *bla*_HMB-1_ and *bla*_HMB-3_ inserted into the pTOPO plasmids. Purified HMB-1, HMB-3, NDM-1 and IMP-1 were used for steady state kinetic measurements of hydrolysis of selected beta-lactams.

**Results and discussion:**

The isolate was obtained from a tracheostomy wound swab. Susceptibility testing showed that it was resistant to all beta-lactams, except aztreonam; however, no classical carbapenemase genes were identified by routine testing. WGS produced a complete chromosome of 6.1 Mb but no plasmids, and identified a gene encoding a novel MBL, namely HMB-3, differing from HMB-1 by 23 amino acid substitutions. HMB-3 was chromosomally encoded and located within a 25.6 kb genomic island. HMB-3 conferred resistance to most beta-lactam antibiotics, except piperacillin (MIC 4 mg/L), aztreonam (0.125 mg/L) and cefiderocol (2 mg/L). Site-directed mutagenesis of *bla*_HMB-1_ and *bla*_HMB-3_ revealed that a single amino acid change, E181H/H181E, in active site loop 10, could significantly alter the MIC of cefiderocol. HMB-3 demonstrated increased hydrolytic activity against cefiderocol compared with HMB-1 and NDM-1.

**Conclusions:**

Here we described a novel MBL enzyme responsible for acquired resistance to carbapenems and reduced susceptibility to cefiderocol in *Pseudomonas* spp.

## Introduction

Carbapenem-resistant *Pseudomonas* spp. (CRP), particularly those that produce metallo-beta-lactamases (MBLs), represent a threat to public health.^1,2^ Infections caused by CRP are often multidrug resistant, leaving few viable treatment options. This has led to its classification by the World Health Organization as a High Priority pathogen on the 2024 Bacterial Priority Pathogens List.^3^ CRP are usually multidrug resistant due to their propensity to acquire resistance genes in addition to their numerous intrinsic resistance mechanisms (e.g. efflux, permeability defects).^4^ The novel cephalosporin, cefiderocol, was approved by the Food and Drug Administration and European Medicines Agency in 2019 and 2020, respectively, and has been shown to exhibit excellent *in vitro* activity against CRP that are resistant to beta-lactam/beta-lactamase inhibitor (BL/BLI) combinations such as ceftazidime-avibactam, ceftolozane-tazobactam and imipenem-relebactam.^5,6^ Subsequently, the Infectious Diseases Society of America has listed cefiderocol as a preferred treatment option for infections caused by MBL-producing CRP.^7^ However, reduced susceptibility or resistance to cefiderocol has been reported in CRP, usually associated with the production of some beta-lactamases, particularly NDM-type enzymes,^8,9^ and mutations within TonB-dependent iron transporters (e.g. *piuA*, *pirA*) that are used for cefiderocol entry into the bacterial cell.^10^ Currently, there are several novel BL/BLI inhibitor combinations under development, or are not yet approved, that have been reported to exert activity against MBL-producing microorganisms in *in vitro* studies, including aztreonam-avibactam, cefepime-taniborbactam, cefepime-zidebactam and meropenem-xeruborbactam.^11^

Among CRP, the most frequently identified acquired MBLs globally are members of the B1 subclass, namely NDM-, VIM- and IMP-type enzymes, although numerous other members of this subclass have been reported in *Pseudomonas* spp., with distributions often varying geographically (e.g. SPM enzymes predominating in Brazil, but being rare elsewhere).^12^ The B1 subclass Hamburg MBL (HMB)-1 was initially described in 2017 from a clinical *Pseudomonas aeruginosa* isolate, first recovered in 2012 from the rectal swab of a patient hospitalized in Germany.^13^ Since then just one other HMB variant, HMB-2, has been reported in *P. aeruginosa* in the USA.^14^ The HMB-1 variant was described as conferring resistance to broad-spectrum cephalosporins and to the carbapenems imipenem and meropenem, but not to ertapenem, piperacillin and aztreonam.^13^ HMB-1 belongs to the monophyletic group B1.2, along with KHM-like and IMP-like enzymes,^15^ with which it shares roughly 74% and 58% amino acid identity, respectively.

Here, we describe the identification and characterization of a novel HMB variant, HMB-3, found in a *P. asiatica* isolate in Switzerland.

## Materials and methods

### Bacterial isolates and antimicrobial susceptibility testing

A carbapenem-resistant isolate, initially identified as *Pseudomonas putida*, was sent to the Swiss National Reference Centre for Emerging Antibiotic Resistance (NARA) for further investigation. The HMB-1-producing *P. aeruginosa* isolate was obtained from the German National Reference Laboratory for Multidrug-Resistant Gram-negative Bacteria.^13^ Susceptibility testing to beta-lactam antibiotics was performed by disc diffusion, and MICs were determined by broth microdilution according to CLSI guidelines.^16^ Avibactam, taniborbactam, and zidebactam were used at a fixed concentration of 4 mg/L, and xeruborbactam was used at a fixed concentration of 8 mg/L. The concentrations for taniborbactam, zidebactam and xeruborbactam were selected to be in line with those used in previous publications.^17,18^

### WGS

WGS was performed on an Illumina NextSeq1000 instrument as previously described.^19^ Assemblies were performed using the Shovill v.1.1.0 pipeline (https://github.com/tseemann/shovill) and contigs were annotated using Prokka v.1.14.6.^20^ Species identification, sequence types (STs), the presence of resistance genes, and plasmid replicon types were determined using KmerFinder v.3.2, MLST v.2.0, ResFinder v.4.7.2^21^ and PlasmidFinder v.2.1,^22^ on the Center for Genomic Epidemiology platform (https://cge.cbs.dtu.dk/services/).

For long-read sequencing, total genomic DNA (gDNA) was extracted from a bacterial culture grown overnight, using the QIAamp DNA Mini Kit (Qiagen, Valencia, CA, USA) and sequenced using the MinION Mk1B (Oxford Nanopore Technologies, Oxford, UK). Sequencing libraries were prepared using a 1D chemistry Ligation Sequencing Kit (SQK-LSK114; Oxford Nanopore Technologies) and performed on a R10.4.1 Flongle Flow Cell (FLO-FLG114; Oxford Nanopore Technologies). Hybrid assembles, using both short and long-read data, were performed using UniCycler v.0.5.1.^23^

Sequencing reads from this study were deposited in the NCBI Sequence Read Archive under BioProject accession number PRJNA1391036.

### Cloning and site-directed mutagenesis experiments

The *bla*_HMB_ alleles, respectively encoding HMB-1 and HMB-3, were amplified using primer pairs HMB-1-Fw/HMB-1-Rv, HMB-3-Fw/HMB-3-Rv, HMB-1-pUCP-Fw/HMB-1-pUCP-Rv and HMB-3-pUCP-Fw/HMB-3-pUCP-Rv (Table S1, available as Supplementary data at *JAC* Online), and cloned into vectors pCR-Blunt II-TOPO (Invitrogen, ThermoFisher), and pUCP24, before transformation into *Escherichia coli* Top10. Transformants were selected on LB agar plates supplemented with either kanamycin (50 mg/L for pCR-Blunt II-TOPO) or gentamicin (15 mg/L for pUCP24). Successful transformants were confirmed by PCR amplification and sequencing of the alleles. Vectors pUCP24-HMB-1 and pUCP24-HMB-3 were subsequently transformed into *P. aeruginosa* PAO1 by electroporation.

Site-directed mutagenesis was performed using the Q5^®^ Site-Directed Mutagenesis Kit (New England Biolabs) on plasmids pTOPO-HMB-1 and pTOPO-HMB-3 to make the change from glutamic acid to histidine at position 181 (E181H, position relative to HMB-3 amino acid numbering) in pTOPO-HMB-1 and the inverse change in pTOPO-HMB-3. The primers used (HMB-1-E181H-Fw/HMB-1-E181H-Rv and HMB-3-H181E-Fw/HMB-3-H181E-Rv) are listed in Table S1.

### Expression and purification of HMB-1 and HMB-3

The *bla*_HMB-1_ and *bla*_HMB-3_ alleles, both lacking the signal peptide sequence as predicted by SignalP-5.0 (https://services.healthtech.dtu.dk/services/SignalP-5.0/), were amplified by PCR using primers pOPINF-HMB-1-Fw, pOPINF-HMB-1-Rv, pOPINF-HMB-3-Fw and pOPINF-HMB-3-Rv (Table S1), and cloned into the pOPINF vector^24^ featuring a protease-cleavable N-terminal His-tag. The resulting plasmids were transformed into *E. coli* BL21 DE3 (pLysS), and protein expression and purification were performed as previously described.^25^

### Determination of kinetic parameters

Enzyme assays were performed at 25°C in 50 mM HEPES pH 7.5, 150 mM NaCl, 0.02 mM ZnSO_4_ and 50 μg/mL bovine serum albumen in half-area 96-well plates (Greiner), and a CLARIOstar microplate reader (BMG Labtech, Ortenberg, Germany). Kinetic parameters were determined by measuring initial rates of substrate hydrolysis and subsequently analysed using the Michaelis–Menten function in GraphPad Prism version 10.0.0 for Windows (GraphPad Software, Boston, MA, USA, www.graphpad.com). The following wavelengths and extinction coefficients were used: nitrocefin (Δ*ε*485 = 17 420 M^−1^ cm^−1^);^26^ cefepime (Δ*ε*260 = −9970 M^−1^ cm^−1^);^27^ cefiderocol (Δ*ε*259 = −9430 M^−1^ cm^−1^);^8^ ertapenem (Δ*ε*295 = −9970 M^−1^ cm^−1^);^28^ imipenem (Δ*ε*300 = −9000 M^−1^ cm^−1^)^26^ and meropenem (Δ*ε*300 = 9600 M^−1^ cm^−1^).^29^

### IC_50_ measurements

IC_50_ measurements were performed over a range of concentrations for inhibitors taniborbactam and xeruborbactam, as previously described.^30^ Measurements were performed on a Genesys 10S UV/VIS spectrophotometer (Thermo Scientific) spectrophotometer using a wavelength of 262 nm for cephalothin. Enzymes were preincubated with inhibitor for 5 minutes before the addition of cephalothin All measurements were performed in triplicate.

## Results and discussion

### Phenotypic and genotypic profiling of PA342

Isolate PA342 was recovered from a tracheostoma wound swab of a 45-year male. Susceptibility testing showed that the isolate was non-susceptible to all tested beta-lactams (penicillins, cephalosporins, aztreonam and carbapenems) with the exception of cefiderocol (MIC of 1 mg/L) (Table 1). By testing cefepime and meropenem in combination with the zidebactam, xeruborbactam and taniborbactam BLIs, either marginal (2-fold) or no decrease in MICs was observed. Results obtained from the NG-Test^®^ CARBA-5 immunochromatographic test for the most common carbapenemases (KPC, OXA-48-like, NDM, VIM and IMP enzymes) as well as from PCRs for the MBL encoding genes *bla*_NDM_, *bla*_VIM_ and *bla*_IMP_ were all negative. Subsequently, WGS was performed using both Illumina (short-read) and Oxford Nanopore Technologies (long-read) platforms, to allow for further characterization. WGS identified the isolate as *Pseudomonas asiatica*, recently designated as a new species in 2019 and a member of the *Pseudomonas putida* group,^31^ and could be assigned to ST15 according to the *P. putida* MLST scheme.^32^ Analyses using ResFinder^12^ identified the presence of beta-lactamase genes encoding the intrinsic AmpC and the class A carbenicillinase, CARB-2. Since the presence of either of these beta-lactamases did not explain the observed phenotype, a manual examination of the assembled and annotated genome was performed, and this allowed the identification of a protein with an MBL motif. BLAST analyses showed that this protein exhibited closest similarity to HMB-1 and HMB-2 (94% and 90% amino acid identity respectively), hence its designation as HMB-3.

**Table 1.** Susceptibility testing of clinical isolate PA342, and recombinant *E. coli* Top10 strains producing HMB-1, HMB-3 and HMB-3/H181E

	MIC (mg/L)																
Strain	AMX	PIP	CEF	FOX	CTX	CAZ	FEP	FEP-TAN	FEP-ZID	ZID	ATM	ATM-AVI	ETP	IPM	MEM	MEM-XER	FDC
PA342	>256	128	>256	>256	>256	>256	256	256	128	32	16	16	>256	8	128	64	1
EC Top10	4	2	8	2	≤0.125	≤0.125	≤0.125	≤0.125	≤0.125	≤0.125	≤0.06	≤0.06	≤0.06	≤0.06	≤0.06	≤0.25	≤0.06
pTOPO-HMB-1	>256	4	256	>256	32	128	4	4	≤0.125	≤0.125	0.125	0.125	0.5	0.25	0.5	≤0.25	0.25
pTOPO-HMB-3	>256	4	>256	>256	64	256	16	16	≤0.125	≤0.125	0.125	0.125	8	1	4	1	2
pTOPO-HMB- 1/E181H	>256	4	256	256	32	128	4	4	≤0.125	≤0.125	0.125	0.125	1	0.25	0.5	≤0.25	2
pTOPO-HMB- 3/H181E	>256	4	>256	>256	64	256	16	16	≤0.125	≤0.125	0.125	0.125	2	1	4	1	0.5
PAO1	>256	4	>256	>256	16	2	2	2	≤0.125	2	2	2	8	1	0.5	0.5	0.25
PAO1-pUCP24- HMB-1	>256	4	>256	>256	>256	>256	>256	>256	≤0.125	2	2	2	128	2	16	16	4
PAO1-pUCP24- HMB-3	>256	4	>256	>256	>256	>256	>256	>256	≤0.125	2	2	2	256	4	32	32	16

AMX, amoxicillin; PIP, piperacillin; CEF, cephalothin; FOX, cefoxitin; CTX, cefotaxime; CAZ, ceftazidime; FEP, cefepime; FEP-TAN, cefepime-taniborbactam; FEP-ZID, cefepime-zidebactam; ZID, zidebactam; ATM, aztreonam; ATM-AVI, aztreonam-avibactam; ETP, ertapenem; IPM, imipenem; MEM, meropenem; MEM-XER, meropenem-xeruborbactam; FDC, cefiderocol.

### *Phenotypic profiling of recombinant* E. coli *and* P. aeruginosa *strains producing HMB-like MBLs*

Susceptibility testing of recombinant *E. coli* Top10 and *P. aeruginosa* PAO1 strains expressing either the *bla*_HMB-3_ gene from isolate PA342 or the *bla*_HMB-1_ gene, showed that in both strain backgrounds, the HMB-variant genes conferred resistance to most beta-lactams (except piperacillin, aztreonam and cefiderocol), and reduced susceptibility or resistance to the carbapenems (Table 1). The gene sequence of novel variant was therefore submitted to GenBank and was assigned allele number HMB-3 (GenBank accession no. PV035090). Some notable differences were observed between the phenotypes of recombinant *E. coli* strains expressing *bla*_HMB-1_ and *bla*_HMB-3_, namely to cefepime, meropenem and cefiderocol (Table 1). Interestingly, strains producing HMB-1 and HMB-3 exhibited the same MIC values when comparing cefepime and cefepime-taniborbactam (MIC of 16 mg/L), suggesting a lack of significant inhibitory activity of taniborbactam towards HMB enzymes, similar to previous observations for strains producing IMP enzymes.^33^ By contrast, supplementation with the zidebactam and xeruborbactam BLIs significantly reduced the MIC of meropenem and cefepime, respectively. The lack of inhibition by taniborbactam could be explained by structural similarities between IMP and HMB enzymes, in particular the presence of positively charged residues within the active site binding pocket that have been hypothesized to inhibit efficient binding of taniborbactam.^33^ Of note, the inhibitory property of xeruborbactam towards HMB enzymes also mirrors what is observed with IMP-like enzymes. However, in the PAO1 strain background, no reduction in meropenem MICs was observed when combined with xeruborbactam. This observation is in line with previous reports that meropenem-xeruborbactam exhibits limited activity in carbapenemase-producing *P. aeruginosa* strains, largely due to intrinsic efflux mechanisms (e.g. MexAB-OprM) that hinder its effect.^18^ Finally, the reduction of the cefepime MICs when combined with zidebactam probably resulted from the well described very significant antibacterial activity of zidebactam rather than from its capacity to inhibit the hydrolytic activity of the HMB-1 or HMB-3, due to the overall lack of inhibitory action of zidebactam against MBLs as a whole.^34^ This is illustrated by the low MICs determined to zidebactam alone.

A sequence alignment of HMB-1 and HMB-3 identified one amino acid, namely residue 181, that differed between the two variants, with HMB-1 possessing Glu181 and HMB-3 His181. Position 181 is located within active site loop 10, an area where mutations have previously been implicated in beta-lactam resistance in other subclass B1 MBLs.^35^ To gain insight into the discrepant MICs observed between the HMB-1 and HMB-3 producers, site-directed mutagenesis of both *bla*_HMB-1_ and *bla*_HMB-3_ was performed to generate either E181H (*bla*_HMB-1_) or H181E (*bla*HMB-3) substitutions. These changes resulted in increased in MICs for ertapenem and cefiderocol in the HMB-1 E181H mutant and the inverse in the HMB-3 H181E mutant. This suggested that this residue was, at least in part, responsible for the increased MICs observed for these agents in recombinant *E. coli* strains expressing *bla*_HMB-3_, relative to *bla*_HMB-1_.

### Enzyme kinetics

Comparative biochemical characterization of purified recombinant HMB-1 and HMB-3 showed that both enzymes could hydrolyse all substrates tested, including nitrocefin, cefepime, cefiderocol, ertapenem, imipenem and meropenem (Table 2). Comparable *k*_cat_/*K*_M_ values were observed for nitrocefin, cefepime and imipenem (all within 20%), however, HMB-3 exhibited significantly higher catalytic efficiency against cefiderocol, ertapenem and meropenem. Compared with NDM-1, an enzyme known to efficiently hydrolyse cefiderocol,^8,9^ HMB-1 exhibited similar catalytic activity, but HMB-3 exhibited markedly increased (3-fold higher) *k*_cat_/*K*_M_ values. One notable difference was the significantly higher affinity of HMB-3 for cefiderocol, as evidenced by the decrease in *K*_M_ values, compared with NDM-1. Despite both enzymes types belonging to the B1.2 monophyletic group, the HMB enzymes, exhibited significantly greater turnover (*K*_cat_) values for cefiderocol, compared with IMP-1.

**Table 2.** Kinetic parameters of purified HMB-1, HMB-3, NDM-1 and IMP-1

	HMB-1			HMB-3			NDM-1			IMP-1		
Antibiotic	*k*_cat_ (s^−1^)	*K*_M_ (µM)	*k*_cat_/*K*_M_ (M^−1^s^−1^)	*k*_cat_ (s^−1^)	*K*_M_ (µM)	*k*_cat_/*K*_M_ (M^−1^s^−1^)	*k*_cat_ (s^−1^)	*K*_M_ (µM)	*k*_cat_/*K*_M_ (M^−1^s^−1^)	*k*_cat_ (s^−1^)	*K*_M_ (µM)	*k*_cat_/*K*_M_ (M^−1^s^−1^)
Nitrocefin	110.4 ± 4.40	25.9 ± 4.90	4.26 × 10^6^	90.75 ± 4.05	25.76 ± 5.24	3.5 × 10^6^	ND	ND	ND	ND	ND	ND
Cefepime	1.21 ± 0.02	8.89 ± 0.76	1.36 × 10^5^	0.86 ± 0.02	6.42 ± 0.74	1.35 × 10^5^	ND	ND	ND	ND	ND	ND
Cefiderocol	19.86 ± 0.75	90.15 ± 8.03	2.20 × 10^5^	35.42 ± 1.29	51.59 ± 5.82	6.87 × 10^5^	26.8 ± 1.3	121.2 ± 12.55	2.21 ×10^5^	1.0 ± 0.04	154.8 ± 10.95	6.50 × 10^3^
Ertapenem	0.35 ± 0.01	19.39 ± 1.85	1.82 × 10^4^	0.54 ± 0.01	11.85 ± 2.13	4.54 × 10^4^	ND	ND	ND	ND	ND	ND
Imipenem	23.81 ± 0.77	72.75 ± 8.15	3.27 × 10^5^	34.94 ± 0.85	91.41 ± 7.33	3.82 × 10^5^	ND	ND	ND	ND	ND	ND
Meropenem	0.44 ± 0.01	5.23 ± 0.49	8.37 × 10^4^	0.86 ± 0.02	6.42 ± 0.74	1.34 ± 10^5^	ND	ND	ND	ND	ND	ND

ND, not determined.

IC_50_ assays showed no significant inhibitory activity of taniborbactam against HMB-1, HMB-3 or IMP-1, correlating with the previous literature on IMP-1^33^ and the susceptibility testing results in this study (Table 3). However, xeruborbactam was shown to be effective with IC_50_ values of 1.51 and 1.78 for HMB-1 and HMB-3, respectively.

**Table 3.** Inhibitory concentrations of taniborbactam (TAN) and xeruborbactam (XER) against HMB-1, HMB-3, IMP-1 and NDM-1

	IC_50_ (µM)	
Enzyme	TAN	XER
HMB-1	>100	1.78
HMB-3	>100	1.51
IMP-1	>100	1.15
NDM-1	1.07	0.73

### *Genetic environment of* bla*_HMB-3_*

**Figure 1.**
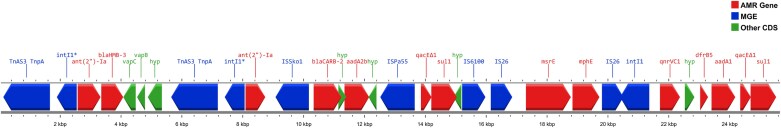
The genetic environment of *bla*_HMB-3_, comprising a 25.6 kb chromosomally encoded resistance island.

The combined Illumina and Nanopore sequencing methods allowed the complete genome of PA342 to be sequenced to closure. No plasmids were harboured by PA342, and the chromosome had a size of 6.1 Mb, with a GC content of 62.3%. The gene encoding HMB-3 was found to be located on the chromosome and was found within a 25.6 kb region comprising multiple genes encoding mobile genetic elements and 12 additional antibiotic resistance genes encoding resistance to aminoglycosides (*aadA1*, *aadA2b* and *ant(2”)-Ia, ant(2”)-*like), fluoroquinolones (*qnrVC1*), sulphamethoxazole [*sul1* (two copies)], trimethoprim (*dfrB5*), macrolides (*mphE*, *msrE*) and beta-lactams (*bla*_CARB-2_ and *bla*_HMB-3_) (Figure 1). The gene encoding HMB-3 was encoded within a class 1 integron, and immediately flanked downstream by the aminoglycoside resistance gene, *ant(2”)*-like, and upstream by genes encoding the VapBC toxin-antitoxin system. In addition, a DUF3592 domain protein exhibiting 97% homology to a protein found in *Cellvibrio chitinivorans* (WP_323813451) was determined. The GC content of the genes encoding the HMB-1, -2 and -3 variants are 43.3%, 44.4% and 43.8%, respectively, suggesting that their origin lies outside of the *Pseudomonas* genus, in which species typically have GC contents in the range of 62%–67%. The significantly lower GC content of 48% observed in *C. chitinivorans* indicates a possible origin. *C. chitinivorans* is a recently described and designated chitinolytic Gram-negative bacterial species, identified from mudflats in China.^36^ BLAST analyses of the HMB-3 protein sequence revealed that *C. chitinivorans* strain NN19 harbours a *bla*_HMB-like_ gene encoding a protein sharing 87% identity to HMB-3, further suggesting that this species may be the origin of the corresponding HMB-encoding genes. However, as only a single *C. chitinivorans* genome is currently available it is not possible to draw any solid conclusions regarding the source of HMB enzymes.

### Conclusions

Here we described HMB-3, a class B1 carbapenemase, including cefiderocol in its hydrolytic substrate profile. The properties of HMB-3 are notable due to the enzymes lack of inhibition by taniborbactam, whereas it was well inhibited by xeruborbactam. similar to its B1.2 counterpart, IMP-1. In contrast to IMP-1, HMB-3 exhibited significantly increased activity against cefiderocol. This observation highlights the divergence in properties and activity, and hence phenotype of strains, expressing genes encoding members of the B1.2 group. Despite the relative rarity of reports of HMB-type enzymes, the continuous monitoring of these and other emerging B1 MBLs is of clinical importance, particularly if activity is observed against important last line antimicrobials, including cefiderocol.

## Supplementary Material

dkag148_Supplementary_Data

## Supplementary data

Table S1 is available as Supplementary data at *JAC* Online.

## References

[1] Tenover FC, Nicolau DP, Gill CM. Carbapenemase-producing *Pseudomonas aeruginosa*—an emerging challenge. Emerg Microbes Infect 2022; 11: 811–4. 10.1080/22221751.2022.2048972 35240944 PMC8920394

[2] Del Barrio-Tofiño E, López-Causapé C, Oliver A. *Pseudomonas aeruginosa* epidemic high-risk clones and their association with horizontally-acquired β-lactamases: 2020 update. Int J Antimicrob Agents 2020; 56: 106196. 10.1016/j.ijantimicag.2020.106196 33045347

[3] World Health Organization (2024) . WHO bacterial priority pathogens list, 2024: Bacterial pathogens of public health importance to guide research, development and strategies to prevent and control antimicrobial resistance. World Health Organisation. https://www.who.int/publications/i/item/9789240093461.

[4] Horcajada JP, Montero M, Oliver A et al Epidemiology and treatment of multidrug-resistant and extensively drug-resistant *Pseudomonas aeruginosa* infections. Clin Microbiol Rev 2019; 32: e00031-19. 10.1128/CMR.00031-19 31462403 PMC6730496

[5] Shields RK, Kline EG, Squires KM et al In vitro activity of cefiderocol against *Pseudomonas aeruginosa* demonstrating evolved resistance to novel β-lactam/β-lactamase inhibitors. JAC Antimicrob Resist 2023; 5: dlad107. 10.1093/jacamr/dlad107 37795425 PMC10546814

[6] Gill CM, Santini D, Nicolau DP et al In vitro activity of cefiderocol against a global collection of carbapenem-resistant *Pseudomonas aeruginosa* with a high level of carbapenemase diversity. J Antimicrob Chemother 2024; 79: 412–6. 10.1093/jac/dkad396 38153232 PMC10832583

[7] Tamma PD, Heil EL, Justo JA et al Infectious Diseases Society of America 2024 guidance on the treatment of antimicrobial-resistant Gram-negative infections. Clin Infect Dis 2024: ciae403. 10.1093/cid/ciae403 39108079

[8] Warecki BA, Tomatis PE, Mojica MF et al Cefiderocol “under siege”? understanding the rise of NDM-mediated resistance to novel agents. Chem Sci 2025; 16: 12519–33. 10.1039/d5sc02122g 40510314 PMC12152739

[9] Warecki BA, Tamma PD, Bonomo RA et al NDM-driven cefiderocol resistance: effect and therapeutic considerations. Lancet Infect Dis 2025; 25: 1068–9. 10.1016/S1473-3099(25)00477-3 40812339

[10] Hardie Boys MT, Pletzer D. A review of recently discovered mechanisms of cephalosporin resistance in *Pseudomonas aeruginosa*. Int J Antimicrob Agents 2025; 66: 107527. 10.1016/j.ijantimicag.2025.107527 40306390

[11] Bassetti M, Vena A, Larosa B et al New antibiotics in clinical pipeline for treating infections caused by metallo-β-lactamases producing Gram-negative bacteria. Curr Opin Infect Dis 2024; 37: 582–8. 10.1097/QCO.0000000000001056 39106036 PMC11556884

[12] Boyd SE, Livermore DM, Hooper DC et al Metallo-β-lactamases: structure, function, epidemiology, treatment options, and the development pipeline. Antimicrob Agents Chemother 2020; 64: e00397-20. 10.1128/AAC.00397-20 32690645 PMC7508574

[13] Pfennigwerth N, Lange F, Belmar Campos C et al Genetic and biochemical characterization of HMB-1, a novel subclass B1 metallo-β-lactamase found in a *Pseudomonas aeruginosa* clinical isolate. J Antimicrob Chemother 2017; 72: 1068–73. 10.1093/jac/dkw554 28065891

[14] Walters MS, Grass JE, Bulens SN et al Carbapenem-resistant *Pseudomonas aeruginosa* at US emerging infections program sites, 2015. Emerg Infect Dis 2019; 25: 1281–8. 10.3201/eid2507.181200 31211681 PMC6590762

[15] Berglund F, Johnning A, Larsson DGJ et al An updated phylogeny of the metallo-β-lactamases. J Antimicrob Chemother 2021; 76: 117–23. 10.1093/jac/dkaa392 33005957

[16] CLSI . Performance Standards for Antimicrobial Susceptibility Testing—Thirty-Third Edition: *M100*. 2023.

[17] Sader HS, Rhomberg PR, Flamm RK et al WCK 5222 (cefepime/zidebactam) antimicrobial activity tested against Gram-negative organisms producing clinically relevant β-lactamases. J Antimicrob Chemother 2017; 72: 1696–703. 10.1093/jac/dkx050 28333332

[18] Le Terrier C, Freire S, Viguier C et al Relative inhibitory activities of the broad-spectrum β-lactamase inhibitor xeruborbactam in comparison with taniborbactam against metallo-β-lactamases produced in *Escherichia coli* and *Pseudomonas aeruginosa*. Antimicrob Agents Chemother 2024; 68: e0157023. 10.1128/aac.01570-23 38727224 PMC11620488

[19] Findlay J, Perreten V, Poirel L et al Molecular analysis of OXA-48-producing *Escherichia coli* in Switzerland from 2019 to 2020. Eur J Clin Microbiol Infect Dis 2022; 41: 1355–60. 10.1007/s10096-022-04493-6 36103096 PMC9556411

[20] Seemann T . Prokka: rapid prokaryotic genome annotation. Bioinformatics 2014; 30: 2068–9. 10.1093/bioinformatics/btu153 24642063

[21] Zankari E, Hasman H, Cosentino S et al Identification of acquired antimicrobial resistance genes. J Antimicrob Chemother 2012; 67: 2640–4. 10.1093/jac/dks261 22782487 PMC3468078

[22] Carattoli A, Zankari E, García-Fernández A et al *In silico* detection and typing of plasmids using PlasmidFinder and plasmid multilocus sequence typing. Antimicrob Agents Chemother 2014; 58: 3895–903. 10.1128/AAC.02412-14 24777092 PMC4068535

[23] Wick RR, Judd LM, Gorrie CL et al UniCycler: resolving bacterial genome assemblies from short and long sequencing reads. PLoS Comput Biol 2017; 13: e1005595. 10.1371/journal.pcbi.1005595 28594827 PMC5481147

[24] Berrow NS, Alderton D, Sainsbury S et al A versatile ligation-independent cloning method suitable for high-throughput expression screening applications. Nucleic Acids Res 2007; 35: e45. 10.1093/nar/gkm047 17317681 PMC1874605

[25] Hinchliffe P, González MM, Mojica MF et al Cross-class metallo-β-lactamase inhibition by bisthiazolidines reveals multiple binding modes. Proc Natl Acad Sci U S A 2016; 113: E3745–54. 10.1073/pnas.1601368113 27303030 PMC4932952

[26] Carenbauer AL, Garrity JD, Periyannan G et al Probing substrate binding to metallo-beta-lactamase L1 from *Stenotrophomonas maltophilia* by using site-directed mutagenesis. BMC Biochem 2002; 3: 4. 10.1186/1471-2091-3-4 11876827 PMC77373

[27] Crowder MW, Walsh TR, Banovic L et al Overexpression, purification, and characterization of the cloned metallo-beta-lactamase L1 from *Stenotrophomonas maltophilia*. Antimicrob Agents Chemother 1998; 42: 921–6. 10.1128/AAC.42.4.921 9559809 PMC105568

[28] Toth M, Vakulenko V, Antunes NT et al Class A carbapenemase FPH-1 from *Francisella philomiragia*. Antimicrob Agents Chemother 2012; 56: 2852–7. 10.1128/AAC.00223-12 22450977 PMC3370778

[29] Calvopiña K, Hinchliffe P, Brem J et al Structural/mechanistic insights into the efficacy of nonclassical β-lactamase inhibitors against extensively drug resistant *Stenotrophomonas maltophilia* clinical isolates. Mol Microbiol 2017; 106: 492–504. 10.1111/mmi.13831 28876489

[30] Mueller L, Masseron A, Prod’Hom G et al Phenotypic, biochemical and genetic analysis of KPC-41, a KPC-3 variant conferring resistance to ceftazidime-avibactam and exhibiting reduced carbapenemase activity. Antimicrob Agents Chemother 2019; 63: e01111-19. 10.1128/AAC.01111-19 31527032 PMC6879233

[31] Tohya M, Watanabe S, Teramoto K et al *Pseudomonas asiatica* sp. nov., isolated from hospitalized patients in Japan and Myanmar. Int J Syst Evol Microbiol 2019; 69: 1361–8. 10.1099/ijsem.0.003316 30810522

[32] Ogura K, Shimada K, Miyoshi-Akiyama T. A multilocus sequence typing scheme of *Pseudomonas putida* for clinical and environmental isolates. Sci Rep 2019; 9: 13980. 10.1038/s41598-019-50299-6 31562354 PMC6765009

[33] Drusin SI, Le Terrier C, Poirel L et al Structural basis of metallo-β-lactamase resistance to taniborbactam. Antimicrob Agents Chemother 2024; 68: e0116823. 10.1128/aac.01168-23 38063400 PMC10848773

[34] Moya B, Barcelo IM, Bhagwat S et al WCK 5107 (Zidebactam) and WCK 5153 are novel inhibitors of PBP2 showing potent “β-lactam enhancer” activity against *Pseudomonas aeruginosa*, including multidrug-resistant metallo-β-lactamase-producing high-risk clones. Antimicrob Agents Chemother 2017; 61: e02529-16. 10.1128/AAC.02529-16 28289035 PMC5444176

[35] Carnevale MC, Palacios AR, Hinchliffe P et al Active site loops of membrane-anchored metallo-β-lactamases from environmental bacteria determine cephalosporinase activity. Antimicrob Agents Chemother 2025; 69: e0191824. 10.1128/aac.01918-24 40548716 PMC12326980

[36] Xin Y, Qin X, Zhang H et al *Cellvibrio chitinivorans* sp. nov., a chitinolytic bacterium isolated from an intertidal mudflat. Int J Syst Evol Microbiol 2025; 75: 006827. 10.1099/ijsem.0.006827 40577047 PMC12281832

