# Supplementary material for: Phenotypic and genotypic characterization of HMB-3, a metallo-beta-lactamase from Pseudomonas asiatica
Source: J Antimicrob Chemother. 2026 May 6;81(6):dkag148. doi: 10.1093/jac/dkag148 (PMC13148013; doi:10.1093/jac/dkag148)
Supplement: dkag148_Supplementary_Data [file dkag148_supplementary_data.docx]

| **Primer** | **Sequence (5’-3’)** | **Purpose** |
| --- | --- | --- |
| HMB-1-Fw | AATCGCACGAATAGGAAAACAAA | Cloning of *bla*_HMB-1_ into pCR-Blunt II-TOPO. |
| HMB-1-Rv | CGGGAGGCCTGGGTATTAAT |  |
| HMB-3-Fw | GCAGATTTAGCACCTGAGTTTT | Cloning of *bla*_HMB-3_ into pCR-Blunt II-TOPO. |
| HMB-3-Rv | TTTGAGCGCATTGAAGGGTT |  |
| HMB-1-pUCP-Fw | GATGATGAGCTCAATCGCACGAATAGGAAAACAAA | Cloning of *bla*_HMB-1_ into pUCP24. |
| HMB-1-pUCP-Rv | GATGATGGATCCCGGGAGGCCTGGGTATTAAT |  |
| HMB-3-pUCP-Fw | GATGATGAGCTCGCAGATTTAGCACCTGAGTTTT | Cloning of *bla*_HMB-3_ into pUCP24. |
| HMB-3-pUCP-Rv | GATGATGGATCCTTTGAGCGCATTGAAGGGTT |  |
| HMB-1-E181H-Fw | TGTAAAACCCCATGGGCTGGGAAATTTAAG | To introduce E181H change in HMB-1 by site-directed mutagenesis. |
| HMB-1-E181H-Rv | AAACAGCCACCGAATAG |  |
| HMB-3-H181E-Fw | TGTAAAACCCGAAGGATTGGGAAATTTAAG | To introduce H181E change in HMB-3 by site-directed mutagenesis. |
| HMB-3-H181E-Rv | AAACAGCCGCCGAATA |  |
| pOPINF-HMB-1-Fw | AAGTTCTGTTTCAGGGCCCGGAAGAACCGCTACCCGAAC | Cloning of *bla*_HMB-1_ into expression vector pOPINF for protein purification. |
| pOPINF-HMB-1-Rv | TGGTCTAGAAAGCTTTATTTCTTCTTTGCAACCGC |  |
| pOPINF-HMB-3-Fw | AAGTTCTGTTTCAGGGCCCGGAAGAACCTCTTCCTGAGCTG | Cloning of *bla*_HMB-3_ into expression vector pOPINF for protein purification. |
| pOPINF-HMB-3-Rv | TGGTCTAGAAAGCTTTATTTTTTCTCCGCAACTGC |  |

**Table S1.** The primers used in this study.
